# Beyond pediatrics: noninvasive spinal neuromodulation improves motor function in an adult with cerebral palsy

**DOI:** 10.1186/s42234-023-00133-2

**Published:** 2024-01-03

**Authors:** Rahul Sachdeva, Kristin Girshin, Yousef Shirkhani, Parag Gad

**Affiliations:** 1 SpineX Inc, Los Angeles, CA 91324 USA; 2 Department of Medicine, International Collaboration On Repair Discoveries (ICORD), University of British Columbia, Vancouver, BC V5Z1M9 Canada; 3 GirshinPT Rancho, Cucamunga, CA 91701 USA

**Keywords:** Spinal Cord Neuromodulation, Noninvasive Stimulation, Cerebral Palsy, Sensorimotor Function

## Abstract

Regaining motor function in individuals with cerebral palsy (CP) has been predominantly studied in children, resulting in an underrepresentation of adults in research efforts. We tested the efficacy of noninvasive spinal neuromodulation with neurorehabilitation (Spinal Cord Innovation in Pediatrics; SCiP™ therapy). A 60-year-old CP participant underwent 8 weeks of SCiP™ therapy, resulting in significant motor recovery measured by 14.2-points increase in gross motor function measure (GMFM-88) score, ~ three times the Minimal Clinically Important Difference (MCID) of 5-points. This represented gains in kneeling, sitting, and walking functions. The improvement in GMFM-88 score was maintained above the MCID at the follow up visit (10.3 points above the baseline), twenty weeks following the last therapy session, indicating a persistent effect of the therapy. Our preliminary findings support the therapeutic promise of SCiP™ therapy for enhancing motor function in CP adults. Broader investigations are needed to establish its wider applicability.

## Background

Cerebral palsy (CP) is a complex neurodevelopmental disorder characterized by motor impairment as a result of non-progressive brain injury during early development (Rosenbaum et al. 2007). Traditionally deemed as a pediatric condition, majority of the research and resources for CP have been directed towards children, leading to a substantial body of literature and a well-established framework for managing childhood cases. However, with advancements in medical care and technology, the life expectancy in individuals with CP has reached closer to general population (Colver et al. 2014; Strauss et al. 2007). This transition deserves increased attention to the unique challenges faced by adults with CP. Individuals ageing with CP are more susceptible to the development of secondary medical conditions, such as musculoskeletal problems, chronic pain, visual and respiratory complications, which can further impair their overall well-being, participation in treatment programs and quality of life (Yi et al. 2019; Turk 2009).

A community-based survey of over 20,000 individuals reports that participation in physical therapy (PT) is negatively associated with aging, as well as comorbidities (Freburger and Holmes 2005). Given that adults with CP undergo a faster decline in physical function and mobility compared to the non-CP aging population, (Yi et al. 2019) it is unsurprising that standard of care therapies (e.g., PT, pharmacological interventions, selective dorsal root rhizotomy, and intramuscular OnabotulinumtoxinA injections) (Damiano 2009; Choi et al. 2019; Lundkvist Josenby et al. 2009; Heinen et al. 2010) exhibit limited impact on improvement in sensorimotor function of aging individuals with CP. This limitation is likely attributed to comorbid factors such as muscle contractures, (Mathewson and Lieber 2015) joint stiffness (Alhusaini et al. 2010) and other age-related conditions that exacerbate rapidly over time in CP population. Another significant drawback of the currently available treatments is their emphasis on symptom management, rather than addressing the underlying neurological cause. This can be a greater challenge in adults who are more likely to have reduced central nervous system plasticity (Burke and Barnes 2006; Johnston 2004).

Spinal cord neuromodulation has emerged as a novel promising tool to improve motor function in various neurological conditions. Over the last decade, we have developed and thoroughly investigated noninvasive spinal cord neuromodulation, delivered transcutaneously via electrodes placed on the skin over the vertebral column (Zhong et al. 2022; Kreydin et al. 2022; Inanici et al. 2018; Gerasimenko et al. 2018; Gerasimenko et al. 2015a). Further, multiple studies have successfully demonstrated that transcutaneous spinal neuromodulation changes the level of excitability of the neural networks resulting in improved voluntary control of movement. We have demonstrated that transcutaneous stimulation can lead to recovery of lower extremity, (Gerasimenko et al. 2015b) upper extremity, (Inanici et al. 2018; Gad et al. 2018a) trunk, (Rath et al. 2018) cardiovascular (Sachdeva 2021) and lower urinary tract function (Havton et al. 2019; Gad et al. 2018b) after SCI. Similar results of improved bladder function have been observed in individuals with Stroke and Multiple sclerosis (Kreydin et al. 2022; Kreydin et al. 2020).

Pertinent to the present study, our pilot trials in 22 children with CP have shown that 8 weeks of noninvasive spinal neuromodulation using SCiP™ device (SpineX Inc., Los Angeles, USA) during PT (termed as SCiP™ therapy) produced a significant improvement in gross motor function [13-point increase in Gross Motor Functional Measure (GMFM-88)], considerably greater than the present standard of care treatments such as PT alone (Hastings et al. 2022; Sachdeva, et al. 2023) It is noteworthy that a 13- point increase in GMFM-88 score denotes a clinically-relevant improvement, compared to the minimal clinical important difference score (MCID) for GMFM-88 at 5 points (Storm et al. 2020). MCID is the gold standard to measure clinical relevance. MCID defines the smallest improvement that a patient can perceive as beneficial (Jaeschke et al. 1989). Further, these functional gains are maintained for at least a few weeks demonstrating the functional neuroplasticity induced in the brain and spinal neural networks. Here we present the case of an adult with CP, who underwent the SCiP™ therapy with PT. The objective of the study was to assess improvement in voluntary sensorimotor function with SCiP™ therapy during PT as measured on the GMFM-88 scale.

## Methods

**Fig. 1:**
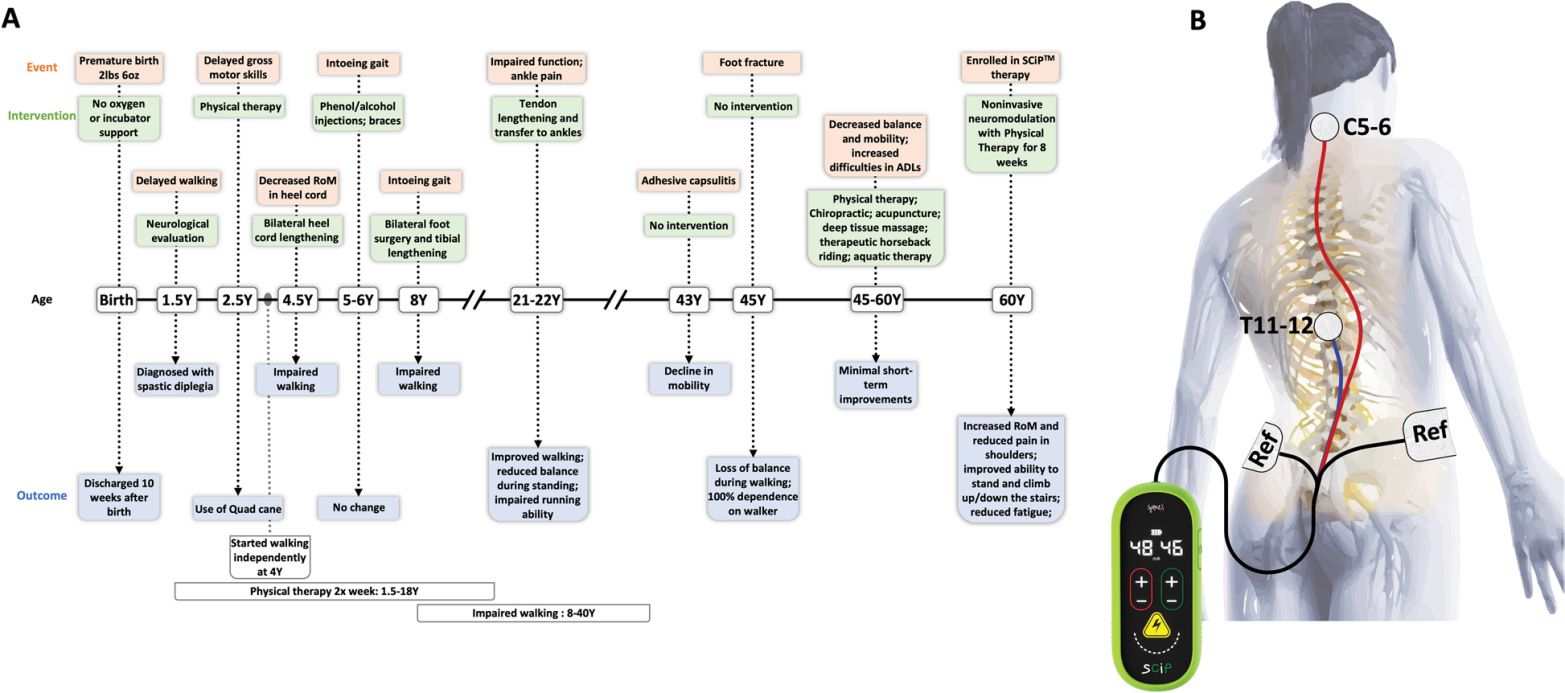
Medical history of the participant and depiction of SCiP™ device. **A** A timeline of events is shown to highlight important therapeutic milestones in attempt to improve motor function prior to enrollment in current trial. Key events include three surgical interventions, long-term enrollment in physical therapy (ages 1.5 to 18 years and 45 to 60 years) and use of assistive devices (e.g., quad cane or braces). **B** Schematic illustration of an adult participant with the SCiP™ device. (ref- reference electrode)

A 60 years old female with Gross Motor Function Classification System (GMFCS) level III CP, (Rosenbaum et al. 2008) was recruited for the study. At the time of recruitment, the participant complained of decreased balance and mobility, resulting in consistently increased difficulties in performing the activities of daily living (ADLs). Prior to enrollment in the trial, the participant was engaged in a number of therapeutic interventions (e.g., physical therapy, chiropractic, acupuncture, deep tissue massage, therapeutic horseback riding and aquatic therapy) for 15 years (i.e., age 45 to 60). All these therapies resulted in minimal, short-term improvements and only marginal improvement in quality of life. Furthermore, the participant also underwent three corrective musculoskeletal surgical interventions during childhood and early adulthood in order to improve gait and reduce pain. A detailed medical history is described in Fig. 1A. At the time of enrollment in the study, the participant was taking oxybutynin once per week that was continued throughout the trial and the follow up period.

The study participant provided informed consent to participate in the investigational study and for the data and images to be shared. In the present study, the participant received 8 weeks of SCiP™ therapy during PT (two 60 min sessions per week, Monday and Wednesday mornings) delivered by a trained physical therapist using the proprietary SCiP™ device (Fig. 1B). The spinal neuromodulation was administered using two adhesive electrodes (1.25" in diameter) placed between C5-6 and T11-12 vertebral levels serving as the cathodes, and two adhesive electrodes (3 × 5″) over bilateral iliac crests as reference electrodes. Neuromodulation was delivered using a delayed biphasic waveform paired with a carrier pulse (10 kHz), with 1 µs delay between the positive and negative phases. The delayed biphasic carrier (10 kHz) was combined with a low frequency (30 Hz) burst with a pulse width of 1 ms. The neuromodulation intensity was set at a suprasensory, but sub-motor threshold (~ 20% below the motor threshold. The threshold was determined by a visible motor contraction of abdominal or upper/lower extremities muscles, or an involuntary movement induced by the stimulation) and was well tolerated by the participant. The stimulation ranged from 6-20 mA for C5-6 and 8-22 mA for T11-12, depending on the activity being performed. The PT activities involved sit-to-stand transitions and reaching, side-stepping over level ground, half kneel to standing and treadmill stepping. The detailed PT protocol has been described previously (Hastings et al. 2022; Sachdeva, et al. 2023; Girshin 2023).

Motor function was assessed at baseline and after 8 weeks of therapy using the Gross Motor Function Measure-88 (GMFM-88) (Russell, et al. 2002). GMFM-88 is a validated observational instruments designed to measure change in gross motor function over time using a 4-point Likert scale for 88 items across five dimensions: (A) lying and rolling, (B) sitting, (C) crawling and kneeling, (D) standing, and (E) walking, running, and jumping. Furthermore, the mobility and risk of falling was also assessed at every session using timed up and go (TUG) test. During TUG test, the participant was asked to (1) stand up from the chair, (2) walk at her normal pace to a line on the floor 10 feet away, (3) turn, (4) walk back to the chair at her normal pace, and (5) sit down again.

## Results and discussion

**Fig. 2:**
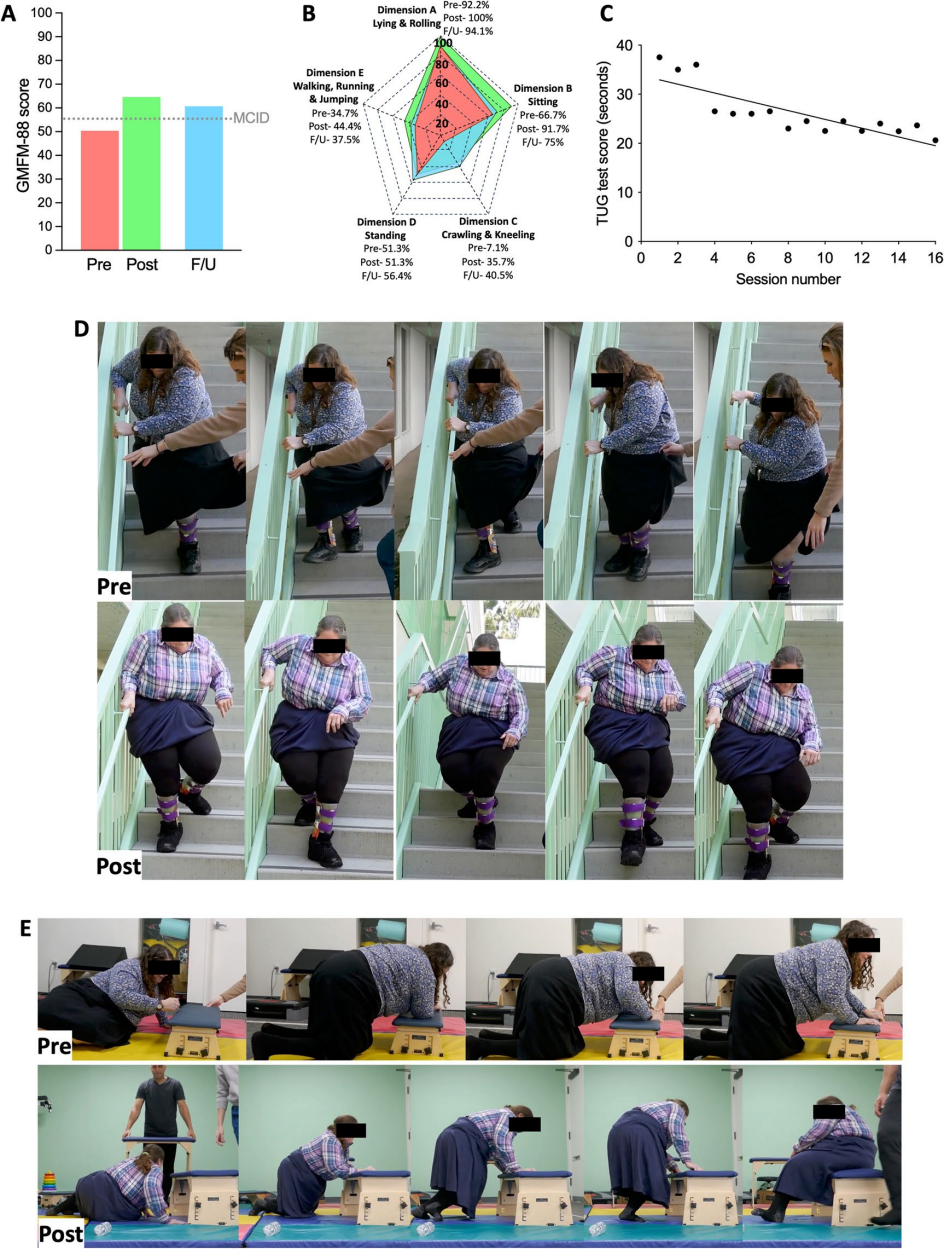
8 weeks of SCiP™ therapy improves motor function. **A** GMFM-88 scores show the motor function at baseline, an increase of 14.2 points post SCiP™ therapy, and a 10.3 point sustained increase from baseline at twenty week follow up. **B** Individual dimension scores show greatest change in dimensions C (crawling and kneeling; 28.6% increase), B (sitting; 25% increase), E (walking, running and jumping; 9.7% increase), and A (lying and rolling; 7.8% increase); with sustained recovery observed in dimension C. **C** time needed to complete TUG test was also reduced as the therapy progressed (nonlinear regression; slope = -0.9; *r*^2^ = 0.64). **D** Demonstration of performance in the use of stairs where SCiP™ therapy improved the participant’s ability to go up and down the stairs with increased confidence and using only one hand on rail (going upstairs not shown). **E** Demonstration of improved ability to kneel and transition from floor to bench, which was not possible before therapy. *It is important to note that all assessments were performed in the absence of an active, real-time SCiP™ therapy*

Prior to enrolling in the study, the participant was able to sit but faced significant difficulty in maintaining balance while reaching to pick up objects. Participant was unable to walk or climb stairs without assistance. The participants functional performance on the GMFM-88 score at baseline (50.4 points) was consistent with a 16- to 18-year-old living with GMFCS level III CP (Hanna S.E. et al. 2008). At the end of 8 weeks of therapy, the participant showed 14.2 points increase in the GMFM-88 score (50.4 pre vs. 64.6 post therapy; Fig. 2A), greater than the MCID = 5 points (Storm et al. 2020). Dimension-specific percentage scores (Fig. 2B) showed greatest change in dimensions C (crawling and kneeling; 28.6% increase; 7.1% pre vs. 35.7%% post therapy), B (sitting; 25% increase; 66.7% pre vs. 91.7% post therapy), E (walking, running and jumping; 9.7% increase; 34.7% pre vs. 44.4% post therapy), and A (lying and rolling; 7.8% increase; 92.2% pre vs. 100% post therapy). No change was observed in standing function measured in dimension D (51.3% pre vs. 51.3% post therapy). Over the course of 8 weeks (16 sessions), the time needed to complete TUG test was also reduced (37.5 s in session 1 vs. 20.6 s in session 16; Fig. 2C; slope = -0.9; *r*^2^ = 0.64). No adverse events were reported during or after the completion of therapy.

Qualitatively, these results translated into substantial improvements in ability to perform ADLs (Fig. 1C and D). The participant was able to go up and down the stairs with increased confidence and using only one hand on rail (Fig. 2D). Participant also demonstrated the ability to get onto hands and knees and transition from floor to bench, which was not possible before therapy (Fig. 2E). Participant also reported increased balance while sitting and bending over to pick up objects and increased range of motion in shoulders (not shown). Although not directly tested, the participant self-reported increase in energy and reduced fatigue during physical activities, as well as improved sleep due to newly-gained ability to reposition in bed.

Twenty weeks following the last therapy session, the motor function was reassessed using the GMFM-88 score. The motor recovery achieved using SCiP™ therapy was maintained, with GMFM-88 score at 60.7 (i.e., > twice the MCID compared to baseline; Fig. 2A, B), primarily reflecting sustained motor recovery in dimensions C (crawling and kneeling).

We present the first evidence of noninvasive spinal neuromodulation-mediated functional recovery in an ageing adult with CP. Although preliminary, the magnitude of resulting improvement (~ three times the MCID) in the presented findings indicate a significant potential in noninvasive spinal neuromodulation with PT as a potential therapeutic strategy in ageing CP population, which poses a greater clinical challenge for functional improvement compared to children. Since individuals with CP spend 76–99% of their waking hours being sedentary, (Verschuren et al. 2016) they are at a greater risk of deterioration of motor performance (Morgan and McGinley 2014) and developing chronic health conditions that may further reduce participation in therapy as they age (Yi et al. 2019). In order to achieve motor learning based on neural plasticity, it has been suggested that optimal rehabilitation should include high intensity, long-lasting training with emphasis on active participation, (Nielsen et al. 2015) which may not be feasible in ageing CP population. This highlights the need for developing interventions enabling participants to perform tasks more effectively. In addition, the neuroplasticity may be further enhanced due to the excited state of the spinal and supraspinal networks. Noninvasive spinal neuromodulation (i.e., SCiP™ therapy) provides targeted, sub-motor threshold electrical pulses which putatively transforms the spinal neural networks into a more active (pro-plasticity) state, which is more receptive to activity-dependent mechanisms (i.e., PT). With majority of research in CP being targeted towards children, only a handful of studies have investigated physical activity interventions in adults with CP (Huche Larsen et al. 2021; Hutzler et al. 2013). One study in adults with CP showed that 12 weeks of physical activity improved the GMFM-88 scores by 5 points, with effects maintained at follow up testing after 1 month (Huche Larsen et al. 2021). Another study showed that while 12 weeks of strength training improves hand and wrist strength in adults with CP, the improvement is lost when the study is discontinued (Hutzler et al. 2013). Our recent work in children with CP shows that the effects of SCiP™ therapy are maintained for at least 8–10 weeks (Sachdeva, et al. 2023). Here we show that the improvement in motor function is maintained beyond 20 weeks, suggesting targeted neural plasticity driven by noninvasive neuromodulation. Another notable distinction in the present study is the age of the participant, compared to the those enrolled in previous studies (60 years vs. mean age of 36 (Huche Larsen et al. 2021) and 46.8 (Hutzler et al. 2013) years). This age difference is likely to pose greater clinical challenges, adding further significance to the present findings. In addition, while neither the MCID nor the average GMFM-88 scores for different age groups have been defined for adults with CP, we envision that the 14-point increase holds greater value for an adult compared to a child with level III CP. Finally, the clinical improvement observed in the 60-year participant directly translates to significant improvements activities of daily living and quality of life. While the observed results are encouraging, it is important to note that they have been tested in a single individual. Therefore, further investigations with a larger sample size are warranted to draw more robust and conclusive outcomes.

## Conclusion

We present the first report demonstrating the efficacy of a noninvasive neuromodulation in improving motor function in an ageing adult with CP. The magnitude of the observed improvement significantly surpasses that reported by current standard-of-care interventions and may potentially exhibit a more enduring effect. Larger and more comprehensive studies are warranted to thoroughly evaluate the potential of this promising therapeutic approach.

## Data Availability

The datasets used and/or analysed during the current study are available from the corresponding author on reasonable request.

## Abbreviations

ADLs: Activities of Daily Living
BRP: Brain Recovery Project
CP: Cerebral Palsy
GMFCS: Gross Motor Function Classification System
GMFM-88: Gross Motor Function Measure-88
MCID: Minimal Clinically Important Difference
PT: Physical Therapy
SCiP: Spinal Cord Innovation in Pediatrics
TUG: Timed up and go
